# Distinct effector functions and synergy of CAR mRNA-engineered T cells and macrophages in the clearance of CD19^+^ leukemia cells

**DOI:** 10.1016/j.jare.2026.01.049

**Published:** 2026-01-18

**Authors:** Liangliang Liao, Cheng Lin, Adrian Kuzmanović, Nan Wang, Alexander Jans, Christian Penners, Kim Ohl, Sandra Johnen, Reka Geczy, Martin Rabel, Samuel Clarke, Aruna Balgi, Christian Liedtke, Matthias Bartneck

**Affiliations:** a Department of Internal Medicine III, University Hospital RWTH Aachen, Pauwelsstraße 30, 52074 Aachen, Germany; b Department of Gastric Colorectal Surgery, China-Japan Union Hospital of Jilin University, Changchun, Jilin 130033, China; c Department of Rheumatology and Shanghai Institute of Rheumatology, Renji Hospital, Shanghai Jiao Tong University School of Medicine, Shanghai 200240, China; d Department of General Surgery, Ruijin Hospital, Shanghai Jiao Tong University School of Medicine, Shanghai 200025, China; e Department of Pediatrics, University Hospital RWTH Aachen, Pauwelsstraße 30, 52074 Aachen, Germany; f Department of Ophthalmology, RWTH Aachen University, Aachen, Germany; g Cytiva, 1055 Vernon Drive, Vancouver, BC V6A 3P4, Canada; h DWI – Leibniz Institute for Interactive Materials, Forckenbeckstraße 50, 52074 Aachen, Germany; i Institute of Technical and Macromolecular Chemistry, RWTH Aachen University, Worringerweg 2, 52074 Aachen, Germany

**Keywords:** Chimeric antigen receptor, mRNA, Live-cell imaging, *SIRPA*, Motility, Phagocytosis

## Abstract

**Introduction:**

Chimeric antigen receptor (CAR) technology represents a groundbreaking advancement in gene therapy. While CAR-T cells are approved for specific types of leukemia, the therapeutic potential of CAR-macrophages remains largely unexplored. Additionally, potentially synergistic effects between CAR-T cells and CAR-macrophages in cancer cell clearance are still elusive.

**Objectives:**

This study aimed to investigate the efficiency and mechanisms of human CAR-T cells and CAR-macrophages for clearing cancer cells using co-culture experiments.

**Methods:**

We encapsulated a second generation CD19 CAR mRNA or enhanced green fluorescent protein (*Egfp*) encoding control mRNA into lipid nanoparticles (LNP) using different methods. We transfected human primary T cells or macrophages with these LNP and studied their CAR expression using flow cytometry or confocal microscopy. Killing and phagocytosis assays were conducted to evaluate the impact of CAR-T cells and CAR-macrophages on CD19^+^ NALM6 leukemia and CD19^-^ control cells. Motility analyses of macrophages, T cells, and cancer cells were done using live-cell imaging.

**Results:**

Activated T cells upregulated the low-density lipoprotein receptor (LDLR), resulting in higher CAR surface expression post-transfection than in non-activated T cells. CAR-T cells efficiently induced death of lymphoma cells. CAR-macrophages cleared cancer cells mainly via phagocytosis and CAR mRNA altered the expression of both M1 and M2-associated genes. Interestingly, CAR macrophages also upregulated the *Signal regulatory protein alpha* (*SIRPA*), a receptor of the CD47 ligand expressed by cancer cells, and knockdown of *SIRPA* by siRNA efficiently counteracted this phenomenon. We noted a decrease in the motility of macrophages, T cells, and cancer cells in co-cultures containing CAR-transfected effector cells. Co-culturing CAR-T cells with CAR-macrophages synergistically enhanced tumor cell eradication.

**Conclusion:**

Our findings underscore the superior cancer cell-killing capability of CAR-T cells. Additionally, CAR-macrophages play a crucial role in clearing cancer cells through phagocytosis, thereby synergistically supporting the activity of CAR-T cells.

## Highlights

• CAR-macrophages eliminate cancer cells mainly through phagocytosis.
• CAR-T cells show potent cytotoxicity even at low effector-to-target ratios.
• CAR mRNA-LNPs induce mixed M1/M2 polarization in macrophages.
• SIRPA is upregulated by CAR mRNA-LNPs, while its knockdown boosts phagocytosis.
• Triple co-culture imaging shows reduced motility after CAR mRNA transfection.

## Graphical abstract

**Figure:**
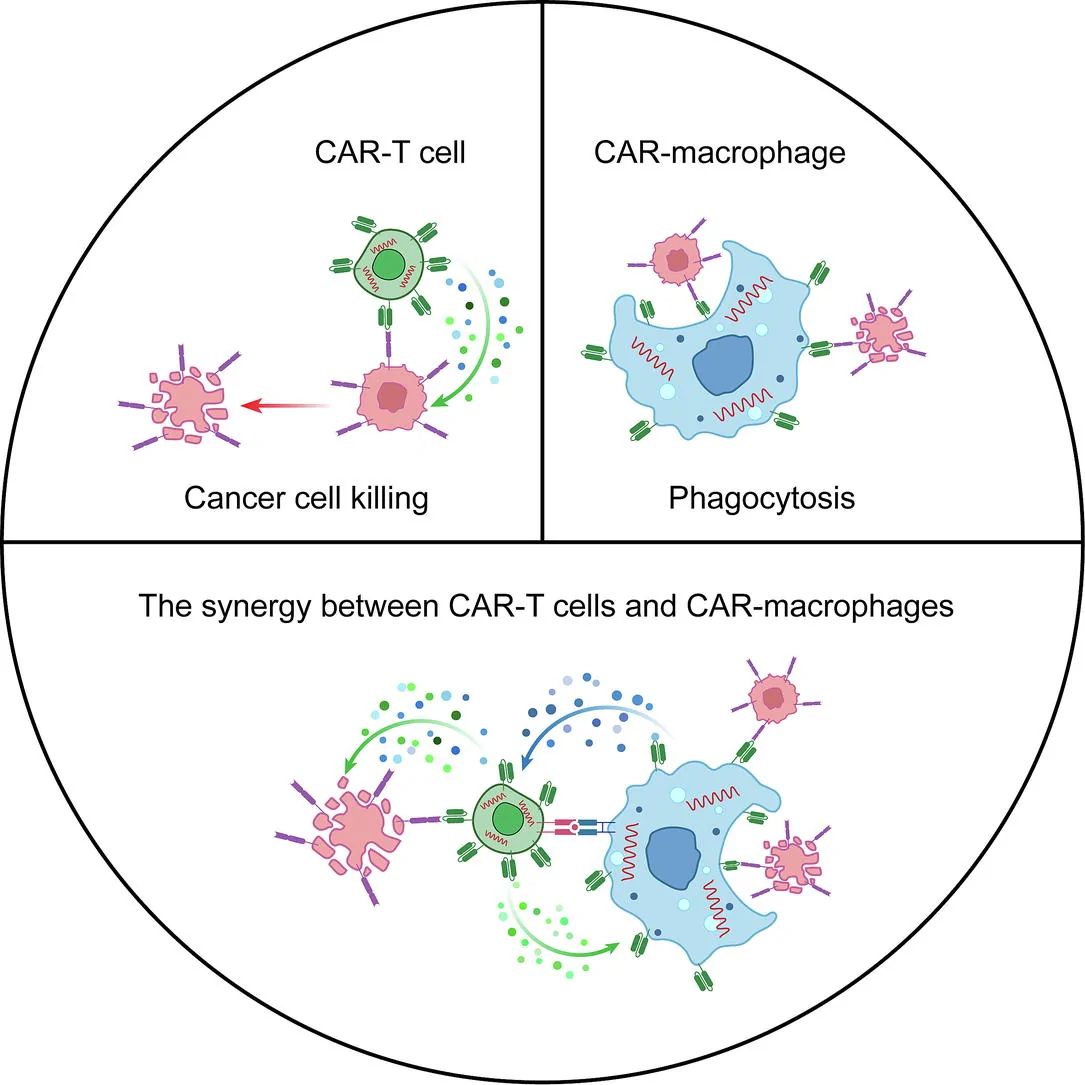

## Introduction

Chimeric antigen receptor (CAR) technology has revolutionized the field of immunotherapy, particularly the treatment of cancer [1]. CAR-T cell therapy involves genetic engineering of patients' own T cells to express receptors that specifically target cancer cells. These modified T cells are then expanded *ex vivo* and infused back into the patient, where they seek and destroy cancer cells. Vector insertion for cell therapy can generally be done using viral or non-viral transfection systems.

Today, all clinically approved CAR-T cell products are manufactured using viral *ex vivo* transduction of autologous T cells [2]. Kymriah (Tisagenlecleucel), manufactured by Novartis, was the first CAR-T cell therapy approved by the U.S. Food and Drug Administration (FDA) in 2017, marking a groundbreaking milestone in cancer immunotherapy. Notably, in late 2025, six CAR-T cell products − Kymriah, Yescarta, Tecartus, Breyanzi, Abecma, and Carvykti − have received approval from the FDA and other authorities. Moreover, there are numerous clinical trials in which also solid tumors are treated with CAR-T cells, such as glioblastoma [3], ovarian cancer, pancreatic cancer, liver cancer, and lung cancer [4].

Non-viral vectors have the capacity to deliver larger genetic payloads and are usually easier to synthesize than viral vectors [5]. Among the non-viral vectors, lipid nanoparticles (LNP) efficiently encapsulate and protect nucleic acids from degradation. LNP have a proven track record of success in clinical settings, most notably in the delivery of messenger RNA (mRNA) vaccines for the SARS-CoV-2 virus, including the Pfizer-BioNTech and Moderna vaccines [6]. mRNA carries transcriptomic information that instructs protein synthesis in living cells. T cells serve as key effectors of antitumor immunity, yet their function is frequently suppressed within the immunosuppressive tumor microenvironment. Enhancing and refining T cell cytotoxicity thus represents a central goal of cancer immunotherapy. Recent studies have demonstrated the successful *in situ* generation of mRNA-encoded CAR-T cells in mice using spleen-targeted ionizable lipid nanoparticles, which conferred potent antitumor activity without the severe cytokine release syndrome typically associated with conventional CAR-T cell therapies [7].

In addition to CAR-T cells, CAR-macrophages (CAR-M) have also been proposed as a cancer therapy option. Macrophages possess robust phagocytic abilities, antigen-presenting functions, can modify the tumor microenvironment, and stimulate adaptive immune responses through the release of cytokines and chemical signals [8], [9]. The dynamic phenotype of macrophages is unique among immune cells and can for instance be altered in response to cytokines, pathogen-associated molecular patterns, metabolic cues, cell–cell interactions, and tissue-specific signals [10]. Macrophages can be classified into two basic sub-types: classically activated M1 macrophages and alternatively activated M2 macrophages. Both types of macrophages play roles in tumour initiation, progression, angiogenesis, and metastasis [11].

Immune cells can trigger the death of cancer cells via different mechanisms. While T cells are capable of killing cancer cells directly via cell–cell contacts, macrophages and dendritic cells (DC) are capable of recognizing “find me” and “eat me” signals emitted by apoptotic cells [12], and as a result neutralize the cells by ingesting them. Klichinsky *et al.* employed an adenoviral vector to generate CAR-M [8], thereby enhancing their anti-tumoral functionality based on phagocytosis. Their study revealed that CAR-M produce pro-inflammatory cytokines and chemokines associated with the M1 phenotype. Furthermore, they demonstrated that a single infusion of human CAR-M reduced tumor burden and prolonged overall survival in mouse models of solid tumors [8]. Pierini and colleagues have recently documented that CAR-M confer long-term anti-tumor immunity [13]. CT-0508, an anti-human epidermal growth factor receptor (HER2) CAR-M therapy in combination with pembrolizumab, is currently the only CAR-M therapy for tumors that has undergone clinical testing (ClinicalTrials.gov ID NCT04660929).

In addition to the well-known checkpoint inhibitors used in lymphoid cell-directed immunotherapy, which block the interaction between the programmed death ligand 1 (PD-L1) expressed by tumor cells and PD1 on lymphoid cells [14], there is a checkpoint between myeloid cells and cancer cells. The interactions between the “don't eat me signal” (CD47) of cancer cells, which functions as a ligand for the receptor *Signal regulatory protein alpha* (*SIRPA*), expressed by myeloid cells, significantly impacts the relationship between macrophages and cancer cells [15].

However, systematic comparisons of human primary CAR-T cells and CAR-M and their interactions remain an unmet need in the field. In this study, we isolated primary human T cells and generated macrophages for transfection with anti-CD19 CAR mRNA-LNP. We used quantitative real-time PCR (qPCR) to study the intracellular presence of the CAR mRNA and applied flow cytometry to verify CAR protein expression at the cellular surface. The efficiency of CAR-T cell-mediated killing and phagocytosis by CAR-M was analyzed by flow cytometry and live-cell imaging. Triple-cell co-cultures were used to investigate the potential synergy and interactions between CAR-M and CAR-T cells in eliminating cancer cells.

## Materials and methods

### Synthetic mRNA and lipids

We used a second-generation CAR mRNA (anti-CD19 scFv from FMC63) comprising a CD8 hinge and transmembrane region followed by intracellular 4-1BB (CD137, Tumor necrosis factor receptor superfamily 9) and T cell receptor Zeta (CD3ζ) signaling domains. A *Thosea asigna* virus 2A (T2A) self-cleaving peptide was placed downstream of the CAR sequence to enable co-expression of the CAR and an EGFP reporter as separate proteins. The CAR cassette was synthesized and subcloned into a pcDNA3.1 vector with a T7 promoter (performed by Creative Biolabs, Shirley, NY, USA). CAR mRNA was synthesized with Cap 1 using CleanCap® AG and enzymatically polyadenylated (TriLink BioTechnologies, San Diego, CA, USA). Therefore, the transfected cells showed surface expression of CAR and intracellular expression of EGFP. The plasmid structure was confirmed by Sanger sequencing. The enhanced green fluorescent protein encoding mRNA (*Egfp* mRNA), L-7601, was also obtained from TriLink.

Heptatriaconta-6,9,28,31-tetraen-19-yl-4-(dimethylamino) butanoate (DLin-MC3-DMA, MC3) was obtained from Hycultec GmbH (Beutelsbach, Germany). The lipids 1,2-distearoyl-*sn*-glycero-3-phosphorylcholine (distearoyl-phosphatidylcholine, DSPC), 1,2-dimyristoyl-*rac*-glycero-3-methoxypolyethylene glycol-2000 (DMG-PEG 2000), and cholesterol were obtained from Sigma-Aldrich (St. Louis, MO, USA). The GenVoy-ILM™ T Cell Kit for mRNA was obtained from Cytiva (Vancouver, Canada).

### Generation of LNP

Small-scale (SS) RNA encapsulation (≤13 µg) into LNP was performed using a NanoAssemblr™ Spark™ nanoparticle formulation system (Cytiva) as reported before [16]. Large-scale (LS) encapsulation of > 100 µg RNA was done using either microfluidic mixing [16], or a NanoAssemblr™ Ignite™ system employing the commercially-available GenVoy-ILM™ T Cell Kit for mRNA, which was used according to the provided protocols and the LNP were stored at −80 °C in freezing buffer until use (both Cytiva). LNP containing co-encapsulated CAR mRNA and *SIRPA* siRNA were produced at a ratio of 2:1. LNP containing siRNA (details shown in Table S3) were generated as reported before and the concentration and encapsulation efficiency of LNP were determined as described earlier [17].

### Characterization of LNP

Dynamic light scattering (DLS) was done to determine the size of LNP, while the zeta potential was measured to assess the surface charge. Both analyses were made using a Zetasizer Nano ZS system (Model: ZEN 3600; Malvern Instruments, Worcestershire, UK). The LNP were diluted 60-fold in PBS for DLS and loaded into cuvettes (ZEN0040, Malvern Panalytical). For zeta potential measurements, the LNP were diluted 100-fold in PBS and transferred into microcuvettes (DTS1070, Malvern Panalytical), as described earlier [16]. The device recorded three measurements of the Z-average size and zeta potential of the particles (Table 1).

**Table 1:** Physicochemical characterization of LNP.

LNP no.	RNA	Mixing technique	Ionizable Lipid	DLS [nm]	Zetapotential [mV]	EE [%]
1 (SS)	*Egfp* mRNA	NanoAssemblr™ Spark™	GenVoy-ILM™ T cell kit	152.3 ± 4.37	−5.98 ± 0.37	93.16
2 (SS)	CAR mRNA	NanoAssemblr™ Spark™	GenVoy-ILM™ T cell kit	140.8 ± 24.72	−6.25 ± 0.59	93.37
3 (SS)	CAR mRNA	Syringe pumps	MC3	129.2 ± 0.47	−3.89 ± 0.33	96.31
4 (LS)	CAR mRNA	NanoAssemblr™ Ignite™	GenVoy-ILM™ T cell kit	142.1 ± 1.31	−6.95 ± 1.19	N/A
5 (LS)	microRNA 26b	Syringe pumps	MC3	125.1 ± 2.04	−10.70 ± 4.95	92.21
6 (LS)	siRNA with a 3′ Alexa Fluor 647 label	Syringe pumps	MC3	129.0 ± 0.30	−4.72 ± 0.99	92.46
7 (LS)	*SIRPA* siRNA	Syringe pumps	MC3	106.8 ± 0.45	−3.58 ± 0.35	94.31
8 (LS)	CAR *+ SIRPA siRNA*	Syringe Pumps	MC3	138.97 ± 2.35	−4.73 ± 0.99	93.46

Table abbreviations: DLS: Dynamic light scattering; SS: small-scale; LS: large-scale; nm: nanometers; mV: milli Volt; EE (%): Encapsulation efficiency (%); *SIRPA*: Signal Regulatory Protein Alpha; MC3: Heptatriaconta-6,9,28,31-tetraen-19-yl-4-(dimethylamino) butanoate.

### Cell isolation and culture

The murine cell lines J774A.1 (ATCC TIB-67), Hepa1-6 (ATCC CRL-1830), NCTC clone 929 (L929 fibroblasts; ATCC CCL-1), and the human cell lines HeLa (ATCC CCL-2), K-562 (ATCC CCL-243), THP-1 (ATCC TIB-202) and human lymphocyte-like blood cancer cells (NALM6) (ATCC CRL-3273) were obtained from the American Type Culture Collection (ATCC, Manassas, VA, USA). Human HeLa cells and the murine cell lines J774A.1, Hepa1-6, and L929 were cultured in Dulbecco’s Modified Eagle Medium (DMEM) from PAN Biotech (Aidenbach, Germany); K-562, THP-1 and NALM6 cells were cultured in Roswell Park Memorial Institute (RPMI)-1640 (PAN Biotech, Aidenbach, Germany). All media were supplemented with 10% fetal bovine serum (FBS), 100 U/mL penicillin, and 100 μg/mL streptomycin. All cells were maintained at 37 °C in a humidified incubator with 5% CO_2_.

Human blood was obtained from the Department of Transfusion Medicine at University Hospital Aachen and used for isolation of peripheral blood mononuclear cells (PBMC) using gradient centrifugation with Pancoll (P04-601000, PAN Biotech). The PBMC were washed with phosphate-buffered saline (PBS) without calcium or magnesium to deplete thrombocytes (indicated by turbid PBS) by centrifugation and adding fresh PBS. Cells were washed by centrifugation and resuspended at three million cells per mL in RPMI-1640 containing 1.5% human autologous serum. We suspended the cells in 2 mL of RPMI-1640 medium and cultured them on small petri dishes (627161, Greiner Bio-one), or in 4 mL on medium-sized petri dishes (628102, Greiner Bio-one). The cells were incubated for 40 min and then the non-adherent cells were removed by washing five times with warm (37 °C) RPMI-1640 medium. The adherent monocytes were then cultured for up to seven days in RPMI-1640 medium supplemented with 5% human autologous serum to differentiate into macrophages. Classical stimulation of macrophages (M1) was done using100 ng/mL of Interferon γ (IFNγ) (300–02, Peprotech) as medium supplement for 24 h, from day five to six of culture.

The Pan T Cell Isolation Kit (130–096-535, Miltenyi Biotec) was used to obtain untouched human T cells from PBMC. T cell activation was performed for 72 h immediately after isolation. We coated 96-well clear round-bottom, tissue culture-treated microplates (353077, Falcon®,) with 30 µL of anti-CD3 monoclonal antibody (clone OKT3, 16–0037-85, Thermo Fisher Scientific) at 5 µg/mL in sterile PBS per well. After 120 min of incubation at 37 °C, the antibody solution was removed, and 50 µL of fresh PBS were added. T cells were then resuspended in TexMACS™ medium (130–097-196, Miltenyi Biotec) supplemented with 100 ng/mL Interleukin-2 (IL-2) (78036, STEMCELL™ Technologies) and 1 µg/mL anti-CD28 monoclonal antibody (16–0289-85, Thermo Fisher Scientific). The T cells were adjusted to a concentration of one million cells per mL, and 200 µL (200,000 cells) were added to each well. The cultures were then incubated for 72 h to induce T cell activation and expansion.

### CAR mRNA transfection, cytotoxicity assessment, and phagocytosis assays

We utilized the Neon Transfection System (MPK5000, Invitrogen) for electroporation-based mRNA transfection according to the instructions of the manufacturer. Briefly, 500,000 activated T cells were transfected with 2 µg of *Egfp* mRNA. Cell viability was assessed 24 h after electroporation using trypan blue staining.

To generate CAR-T cells, activated T cells were transfected with LNP containing CAR or control mRNA for 24 h (from day three to day four) using serum free TexMACS™ cell culture medium (130–097-196, Miltenyi Biotec) supplemented with 1 µg/mL Human Apolipoprotein E (ApoE4) (350–04, PeproTech®).

CAR T cell cytotoxicity assays were conducted using CAR-T cells that were co-cultured with cancer cells in 48-well cell culture plates (677180, Greiner CELLSTAR®). Each well contained 300,000 cells in 300 µL of RPMI-1640 medium supplemented with 5% autologous serum. The ratio of T cells to cancer cells was adjusted to achieve the desired effector to target (E:T) ratios, such as 10:1 (270,000 T cells to 30,000 cancer cells) or 1:1 (150,000 cells each).

CAR-M were generated by transfecting human primary macrophages for 24 h (from day five to six of culture) with 2 µg/mL CAR mRNA-LNP, 1 µg/mL of siRNA-LNP (LNP7, Table 1), or 3 µg/mL of co-encapsulated mRNA and siRNA (LNP8).

The phagocytosis assays were done using adherent CAR-M and floating cancer cells in either 24-well cell culture plates (662160, Greiner CELLSTAR®) or bacterial grade Petri dishes (627161, Greiner Bio-One, 35x10 mm) using RPMI-1640 medium with 5% autologous serum. Macrophages were added to 24-well plates after four days of culture on Petri dishes. In the 24-well format, each well contained 100,000 macrophages in 500 µL medium, while Petri dishes contained one million macrophages in 2 mL medium. Cancer cells were added to achieve specific E:T ratios, for example, for a 10:1 ratio in the 24-well format, we used 90,000 macrophages and 10,000 cancer cells, whereas Petri dishes contained 900,000 macrophages and 100,000 cancer cells.

### Flow cytometry

Flow cytometry was used to check the purity of the isolated cells. On day 0, an aliquot of T cells was used to study T cell purity by flow cytometry. Staining was done as previously described [16], using an APC/Cyanine7-conjugated CD3 antibody (300426, BioLegend), PerCP-Cyanine5.5-conjugated CD4 monoclonal antibody (45–0048-42, eBioscience™), a FITC-conjugated anti-human CD8 antibody (555366, BD Pharmingen™), and an APC-conjugated CD25 antibody (130–114-732, BioLegend).

CD19 CAR detection reagent (130–115-965, Miltenyi Biotec) and APC streptavidin (405207, BioLegend) were used to stain the CAR antigen. PE-conjugated CD19 monoclonal antibody (12–0199-42, eBioscience™) and V500-conjugated CD19 monoclonal antibody (561121, BD Horizon™) were used to assess CD19 expression in the cancer cell lines.

A PE-conjugated CD172a (SIRPα) antibody (130–123-742, Miltenyi Biotec) was used to detect the expression of SIRPα on the surface of human macrophages. PE-conjugated (clone B6H12) antibody (130–123-754, Miltenyi Biotec) or a FITC-conjugated CD47 (clone 2D3) antibody (11–0478-42, eBioscience™) were used to evaluate CD47 surface expression on the cancer cell lines. The B6H12 monoclonal antibody (BE0019-1, BioXCell), which recognises human CD47, was then used to neutralize CD47 on the cancer cells.

To assess apoptosis, the cells were resuspended in 1 × binding buffer (130–092-052, Miltenyi Biotec) and incubated with 1 µg/mL propidium iodide (PI) and FITC-conjugated Annexin V from the Annexin V-FITC Kit (130–092-052, Miltenyi Biotec), in accordance with the manufacturer’s recommendations. As a second approach, the cells were resuspended in 1 × binding buffer (V13246, Invitrogen) and incubated with 1 µg/mL DAPI (62248, Thermo Scientific™) and Alexa Fluor 568-conjugated Annexin V supplied in the Annexin V Kit (A13202, Invitrogen™), in accordance with the manufacturer’s instructions. To assess live and dead cells, either 1 µg/mL PI (P4170, Sigma-Aldrich) or 1 µg/mL Hoechst 33,258 (94403, Sigma-Aldrich) were used.

The synergy assay was based on adding graded numbers of effector cells (0, 20,000, 40,000, 60,000, 80,000, or 100,000 CAR-T cells or CAR-M) into 48-well plates. NALM6 cells were stained with 5 µM CellTrace™ Violet (CTV) (C34571, Invitrogen) and incubated at 37 °C for 30 min in a cell culture incubator. Following the incubation period, the staining reaction was quenched by adding five volumes of RPMI-1640 medium, which was supplemented with 5% fetal calf serum (FCS). After centrifugation at 400 rcf for 10 min, the cell pellet was resuspended in RPMI-1640 medium with 10% FCS and cultured for 24 h in 24-well plates with CAR-T cells and CAR-M. Subsequently, 50,000 CTV-labeled NALM6 cells were added to each well to establish co-cultures. After 24 h, the floating cells were collected for flow cytometric analysis. APC Calibrite beads (340487, BD) served as counting standard to determine the absolute number of remaining NALM6 cells. The bead concentration was first verified using a cell counting chamber. Then, 10 μL of the beads were mixed with 90 μL of the resuspended co-culture cells and analyzed by flow cytometry. The absolute number of NALM6 cells was calculated based on the known bead concentration. Synergy between CAR-T cells and CAR-M was evaluated using the Loewe additivity reference model implemented in SynergyFinder [18].

### Live cell imaging and confocal microscopy

Live cell imaging was performed in 24-well cell culture plates (662160, Greiner CELLSTAR®, Catalog) at 37 °C in a humidified incubator supplied with 5% CO_2_ using an Axio Observer Z1 microscope equipped with an LD Plan-Neofluar 20×/0.4 Korr pH 2 M27 objective, an Axiocam 305 camera and a Colibri 7 LED-light source for fluorescence imaging. Cancer cells were labelled with either CellTracker™ Deep Red dye (CTR) (C34565, Invitrogen), or siRNA with a 3′ Alexa Fluor 647 label encapsulated into LNP (SIR) (Table 2). In triple cell co-culture experiments, 50,000 macrophages per well were transfected for 24 h, while T cells were transfected in the 96-well plates in which they were activated. The co-culture system was then completed by adding 50,000 CAR-T cells and 10,000 CTR-labelled cancer cells to each 24-well. Live-cell imaging was performed at intervals of ten minutes for a total duration of 24 h.

**Table 2:** Fluorescent probes for cancer cell labelling.

Fluorescence Probe	Company	Probe type	Applications	Abbreviation
CellTrace™ Violet	Invitrogen (C34571)	Carboxyfluorescein succinimidyl ester (CFSE)-related	FC	CTV
CellTracker™ Deep Red	Invitrogen (C34565)	Cyanine-based chloromethyl dyes	FC + LCI	CTR
siRNA with 3′ Alexa Fluor 647 label	Qiagen (1027295)	Fluorescently labeled siRNA probes	FC + LCI	SIR

Table abbreviations: FC: Flow cytometry; LCI:Live cell imaging.

Images were exported as TIFF files using ZEN 3.10 software (Zeiss) and subsequently analyzed using ImageJ 1.54 g software. The quantification of phagocytosis and cancer cells was done using the multi-point feature of ImageJ. Cell tracking was done in intervals of ten minutes over 24 h by marking the centre of the nuclei of macrophages, and the centres of T cells, respectively, and cancer cells, across 144 frames. The movement was illustrated by connecting individual movement points. The frame-by-frame annotation of cell centres allowed for the generation of time-lapse sequences, providing a visual representation of cell movement over time. Parameters of cell motility such as track length, and speed were calculated using MTrackJ (ImageJ, NIH, USA).

Confocal microscopy was employed to verify CAR protein translation by human macrophages based on the signal of the EGFP reporter insertion. 50,000 human macrophages were added into ibiTreat µ-Slide 8 Well plates (80826, Ibidi) in 200 µL of RPMI-1640 supplemented with 5% human autologous serum, as described before [16]. The cells were transfected with 2 µg/mL CAR mRNA-LNP 24 h before imaging and were studied for up to 48 h. We used a Zeiss Laser Scanning Microscope (LSM) 980 with Airyscan 2, and the ZEN blue 3.6 software with the LSM Plus module. An LD LCl Plan-Apochromat 40x objective (N.A. 1.2) was used with water immersion. To analyse signals from confocal microscopy, both control and CAR mRNA-LNP macrophages were evaluated using five distinct confocal microscopy images. The EGFP signal was imaged using the 488 nm diode laser with 1.5% laser intensity and the GaAsP-PMT detector gain set to 800 V. The images were post-processed by applying the LSM Plus Processing, the black balance was set to 250 and the white balance set to 6000. The images were exported into ImageJ for further analyses based on the mean fluorescence intensity (MFI).

### Isolation of total RNA

Total RNA was extracted from cell culture samples using a RNeasy Mini Kit (74106, Qiagen) following the manufacturers' instructions, including the optional on-column DNase digestion step. The RNA was converted into cDNA using the High-Capacity cDNA Reverse Transcription Kit (4368814, Applied Biosystems) with the addition of RNase inhibitor (N8080119, Applied Biosystems).

### Gel electrophoresis

Denaturing agarose gels were prepared using agarose (3810.4, Roth) in 3-(N-Morpholino)propanesulfonic acid (MOPS) buffer. The 10X MOPS buffer included 0.4 M MOPS (475922, Sigma Aldrich), 0.1 M sodium acetate (S8750, Sigma Aldrich), and 0.01 M ethylenediaminetetraacetic acid disodium salt dihydrate (EDTA) at pH 7 (E5134, Sigma Aldrich). For electrophoresis, the buffer was diluted to 1X. A 37% formaldehyde solution (F8775, Sigma Aldrich) was added to the gel preparation mixture. RNA Gel Loading Dye (2X) (R0641, Thermo Scientific) was used to load the samples into the agarose gels, and a RiboRuler High Range RNA Ladder (Thermo Scientific, SM1821) was used as a reference marker. The samples were heat-denatured at 65 °C for 10 min, then electrophoresis was performed at 90 V for 90 min.

### Quantitative Realtime-PCR

QPCR was performed in MicroAmp Fast 96-Well Reaction Plates (4346907, Applied Biosystems) and the PowerUp™ SYBR™ Green Master Mix (A25742, Applied Biosystems) was used with 5 ng of cDNA per sample in a QuantStudio 5 thermocycler (Applied Biosystems). Target-specific primers were designed using the NCBI Primer-BLAST tool and obtained from Eurofins. *GAPDH* was used as reference gene, and ΔΔCt data analysis was conducted following the method described by Livak and Schmittgen [19]. All primer sequences used are provided in Table S1. Calculation of the uptake of *Egfp* reporter containing mRNA by cells expressed as parts per million (ppm) related to the total RNA was done as described earlier in detail [16].

### Statistical analysis

Data are expressed as mean ± SD unless stated otherwise. All statistical analyses were done using GraphPad Prism, version 10.1.2 (GraphPad Software, San Diego, CA, USA). Comparisons of measurements between two groups were made using an unpaired or paired two-tailed *t*-test. Statistical difference was assessed using one-way ANOVA and two-way ANOVA for multiple comparisons. P values below or equal to 0.05 were considered statistically significant.

## Results

### Generation of human primary CAR-T cells and CAR-macrophages

T cell activation requires two key signals: the first is the binding of an antigenic peptide/MHC complex to the T cell receptor (TCR), while the second is the co-stimulatory signal that stems from the interaction between the costimulatory receptor CD28 on T cells and its ligands CD80 (B7-1) and CD86 (B7-2) on antigen-presenting cells [20], [21]. Supplementation of the medium with IL-2 stimulates proliferation and survival of T cells during activation [22]. We therefore activated freshly isolated T cells for three days of culture on plates coated with anti-CD3 and supplemented with soluble anti-CD28 antibody and IL-2. The activation of T cells was confirmed by upregulation of the activation marker CD25 and the Low-density lipoprotein receptor (LDLR) (Fig. 1A), which were statistically significant, compared to the expression by non-activated T cells (Fig. 1B).

**Fig. 1:**
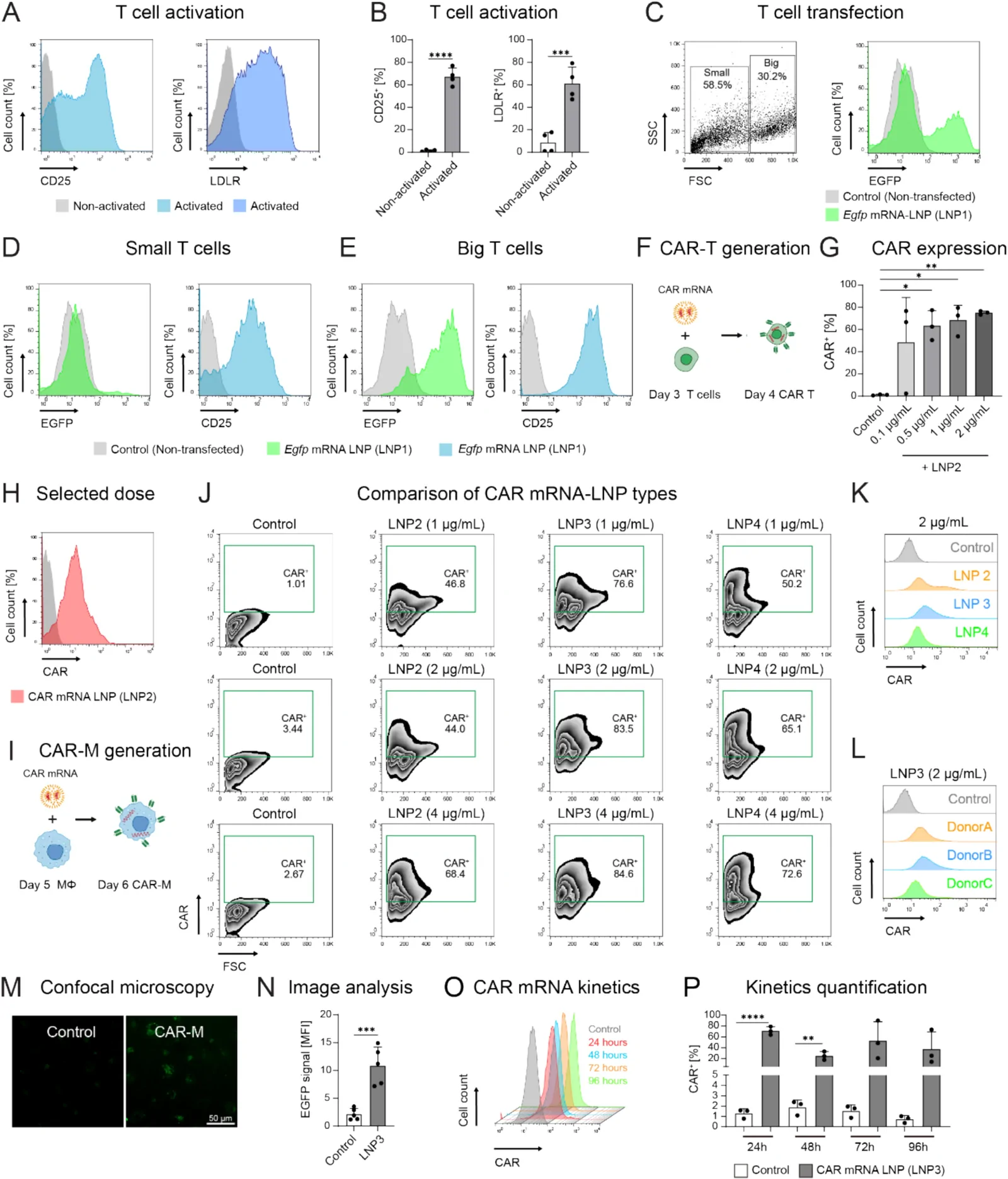
**Translation of *Egfp* and CAR mRNA by human primary T cells and macrophages.** (A) Representative flow cytometric overlays depicting CD25 and LDLR expression in non-activated and activated T cells. (B) Statistical analysis of the data presented in (A) [two-tailed unpaired Student’s *t*-test, n = 4]. (C) Analysis of the morphology (left) of activated T cells and of the EGFP signal using a histogram (right) following transfection with 2 µg/mL *Egfp* mRNA LNP (LNP1) for 24 h. Control T cells were non-transfected but activated T cells. (D) EGFP and CD25 signals of the small and (E) of the big T cells (from C). (F) Scheme for the generation of CAR-T cells. T cells activated from day 0 to day 3 were transfected with 2 µg/mL anti-CD19 CAR mRNA LNP for 24 h. (G) CAR surface staining of control (non-transfected) and CAR mRNA-LNP (LNP2) transfected T cells, and (H) statistical analysis of CAR signals upon transfection with different concentrations of CAR mRNA-LNP (LNP2) [one-way ANOVA with Tukey’s post hoc test, n = 3]. (I) Scheme for the generation of CAR-M. Human primary macrophages at day 5 of culture were transfected with 2 µg/mL anti-CD19 CAR mRNA LNP for 24 h. (J) Representative flow cytometric plots of CAR expression in macrophages transfected with three different concentrations (1, 2, or 4 µg/mL) of three types of CAR mRNA-LNP (details in Table 1), compared to non-transfected control macrophages. (K) CAR expression induced by the three types of LNP (as shown in J) at the concentration of 2 µg/mL. (L) CAR expression of macrophages of three selected donors transfected with LNP3 (2 µg/mL, 24 h transfection). (M) Representative confocal images of non-transfected control (left) and CAR mRNA-LNP (LNP3) transfected macrophages (right) obtained from confocal microscopy, and (N) statistical evaluation of the EGFP signals shown in M [two-tailed unpaired Student’s *t*-test, n = 5]. (O) Representative flow cytometric plots displaying CAR surface expression of control (non-transfected) and CAR-macrophages from 24 to 96 h after transfection, and (P) statistical analysis of the data [two-tailed unpaired Student’s *t*-test, n = 3]. * p ≤ 0.05, ** p ≤ 0.01, *** *p* ≤ 0.001, **** *p* ≤ 0.0001.

Initially, we used electroporation (EP) to deliver *Egfp* mRNA into T cells. Fluorescence microscopy confirmed the translation of the mRNA into EGFP protein (Fig. S1A). To further validate the transfection efficiency and mRNA translation, we performed flow cytometry (Fig. S1B). We found that the EP-based method led to a mean of 78.4% of T cells expressing Egfp mRNA, and to a significantly higher MFI (Fig. S1C). However, compared to untreated and Egfp mRNA LNP-treated cells (LNP1, 2 µg/mL, 24 h transfection), EP reduced the T cell viability from 97% to 49% while transfection with LNP1 led only to a minor reduction of viability (Fig. S1D). The signals obtained from LNP1 transfection were stronger than those obtained from EP, particularly when considering the higher amounts and concentration of mRNA used in EP (Fig. S1E). Therefore, we chose LNP-based transfection for all subsequent experiments.

Upon transfection with LNP1, we noted that the T cells segregated into two distinct populations with different levels of EGFP expression, as apparent in flow cytometric overlays (Fig. 1C). Further analyses revealed distinct EGFP expression levels and activation states in the two T cell populations: the smaller T cell population barely exhibited EGFP expression, with only a fraction of cells showing activation as indicated by CD25 expression (Fig. 1D). In contrast, the larger T cell population demonstrated significantly higher EGFP and very high CD25 expression (Fig. 1E). To further investigate the dose dependency between EGFP expression and LNP concentration, we also examined the EGFP expression 24 h after transfection at 0.1, 0.5, and 1 µg/mL. Flow cytometric analyses confirmed that the larger T cells consistently expressed higher levels of EGFP protein at all selected concentrations (Fig. S2A), with statistically significant differences in EGFP signals at 0.5 and 1 µg/mL (Fig. S2B).

These findings suggest a correlation between cell size, activation state, and transfection efficiency of T cell populations.

After transfecting T cells with CAR mRNA (Fig. S3), we observed that non-activated control T cells displayed minimal surface CAR expression (Fig. S4A). In contrast, flow cytometry revealed a significantly higher fraction of activated T cells expressing CAR, CD25, and LDLR (Fig. S4B). Notably, the MFI of these markers was substantially increased in activated T cells (Fig. S4C), demonstrating not only an increased frequency of expressing cells but also higher expression per cell. Therefore, CAR-T cells were induced using T cells at day three after activation and these were transfected with CAR-mRNA LNP for 24 h (Fig. 1F). We also studied the dose dependency of CAR expression at the concentrations of 0.1, 0.5, 1 µg, and 2 µg/mL of CAR mRNA-LNP and observed that the highest dose of 2  µg/mL of CAR mRNA led to the most consistent protein expression (Fig. 1G), which resulted in a distinct staining of the CAR antigen (Fig. 1H) and therefore, we selected this concentration for upcoming experiments with CAR-T cells.

To evaluate the transfection potential of macrophages, we used macrophages cultured from day 0 to 5 and transfected them for 24 h with CAR mRNA-LNP (Fig. 1I). We compared both in-house formulated and commercially available LNP (LNP2, LNP3 and LNP4, see details in Table 1) for macrophage transfection. Human primary macrophages were transfected with 1, 2, or 4 µg/mL of CAR mRNA-LNP and analysed using flow cytometry at 24 h. All three LNP types yielded comparable efficiency in generating CAR^+^ macrophages at each concentration (Fig. 1J). Representative flow cytometric plots confirmed successful CAR protein expression for all LNP at 2 µg/mL (Fig. 1K). Notably, CAR expression levels differed slightly among macrophages from different donors (Fig. 1L).

In addition, confocal microscopy was utilized to examine the protein translation of the EGFP reporter in the CAR mRNA by CAR-M. It was observed that non-transfected control macrophages exhibited no discernible green fluorescence, while LNP3-transfected macrophages demonstrated a marked increase in EGFP signals (Fig. 1M). This increase was found to be statistically significant (Fig. 1N).

Subsequent studies were conducted to investigate the duration of the CAR surface protein expression by macrophages. We observed that CAR mRNA-transfected macrophages maintained the expression of the CAR protein for a period of 24 to 96 h following the removal of LNP by medium replacement 24 h after the initial transfection (Fig. 1O). However, substantial variability was observed among donors at the 72-hour and 96-hour time points, and CAR protein expression remained significant for up to 48 h (Fig. 1P).

Next, the highest non-cytotoxic concentration of CAR mRNA-LNP for human macrophages was ascertained. Therefore, the cells were exposed to increasing concentrations of LNP4, ranging from 2 µg/mL to 40 µg/mL, for a duration of 24 h. We found that at concentrations of 20 µg/mL or higher, LNP4 resulted in a decreased in viability, as indicated by flow cytometric viability staining (Fig. S5A). This decrease was found to be statistically significant (Fig. S5B).

### Potential off-target effects on other cell types

*In vivo* application of CAR mRNA-LNP is confronted with the challenge that LNP may be taken up by non-target cell types, which could potentially reduce specificity and efficacy. To this end, a comparative analysis was conducted to assess the transfection efficiency, the level of CAR mRNA translation, and *Egfp* reporter expression across a range of cell types, including J774A.1, L929, HeLa, Hepa1-6, human macrophages, and T cells. These cells were then subjected to transfection with 2 µg/mL of CAR mRNA-LNP (LNP3), and subsequent assessment was conducted for their CAR and EGFP reported expression via flow cytometry. We further employed qPCR to meticulously ascertain the uptake of CAR mRNA-LNP by individual cell types.

Flow cytometric analysis revealed varying levels of CAR surface expression across different cell types (Fig. 2A). Quantifications of the flow cytometric signals revealed that human macrophages, with 56.4% CAR^+^ of cells expressing the CAR protein, exhibited the highest expression, followed by 50.2% of T cells. HeLa and L929 cells exhibited moderate expression levels of the CAR, with 36.3% and 31.2% of cells being positive for the receptor, respectively. J774A.1 and Hepa1-6 cells exhibited a conspicuously diminished level of CAR surface expression, with a mere 8.9% and 8.3% of cells being positive for CAR, respectively (Fig. 2B). The MFI of CAR on the surface of various cell types was also analyzed, and the findings from the percentage of CAR^+^ cells were corroborated (Fig. S6A).

**Fig. 2:**
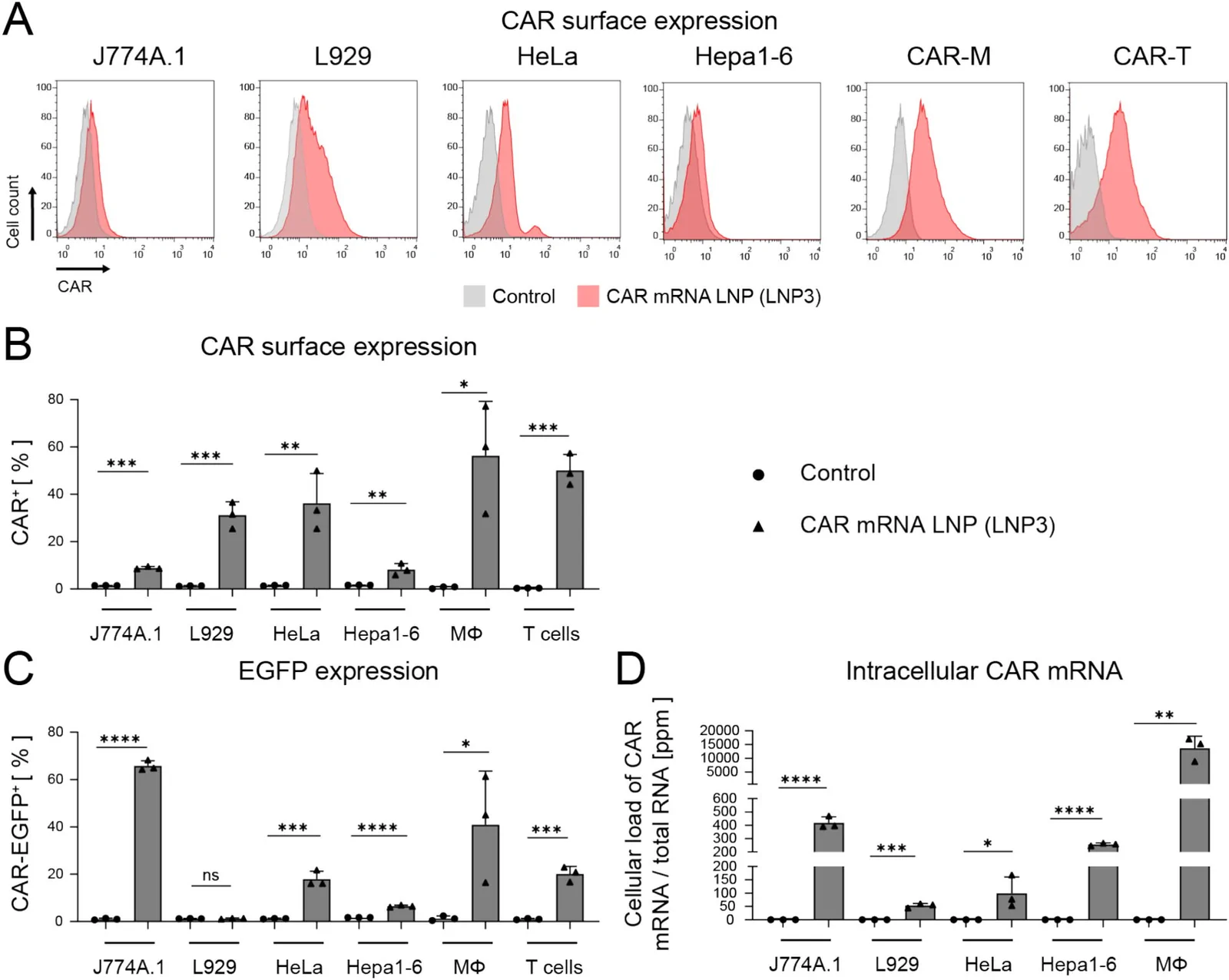
**Uptake and translation of CAR mRNA-LNP by various cell types.** (A) Flow cytometric histograms showing CAR surface staining in non-transfected controls or CAR mRNA-LNP (LNP3) transfected cells (2 µg/mL, 24 h transfection). (B) Flow cytometric analysis showing the percentage of CAR surface expression or (C) 2TA EGFP reporter protein expression by various cell types [two-tailed unpaired Student’s *t*-test, n = 3]. (D) Uptake of LNP3 by the different cell types assessed by *Egfp* qPCR (expressed as parts per million, ppm related to the total RNA) [two-tailed unpaired Student’s *t*-test, n = 3]. * *p* ≤ 0.05, ** *p* ≤ 0.01, *** *p* ≤ 0.001, **** *p* ≤ 0.0001.

Despite their low level of CAR surface expression, J774.A1 cells exhibited the highest level of EGFP reporter expression among all cell types, with 65.8% of cells being EGFP^+^ (Fig. 2C). The human macrophages contained 40.9% EGFP^+^ cells, while only 20.2% of T cells and 18.0% of HeLa cells were EGFP positive. Hepa1-6 cells demonstrated low EGFP expression, with only 6.5% of cells exhibiting positive signals. In contrast, L929 cells exhibited the lowest level of EGFP expression, with only 1.3% of cells displaying positive signals. The analysis of the MFI of EGFP (CAR reporter) across various cell types corroborated the results observed from the percentage of EGFP + cells (Fig. S6B).

Subsequently, we evaluated the 2TA *Egfp* reporter mRNA uptake using qPCR as previously described [16]. In this method, CAR mRNA abundance within cells was quantified by normalizing the intracellular *Egfp* reporter mRNA to the amount of total RNA. We observed that human macrophages internalized the highest amount of CAR mRNA among all tested cell types, with a mean value of 13,693 parts per million (ppm). In comparison, J774A.1 cells incorporated only 419 ppm of CAR mRNA, which was still noticeably greater than the levels measured in L929 (60 ppm), HeLa (77 ppm), and Hepa1-6 cells (257 ppm) (Fig. 2D).

In summary, these findings highlight the remarkable capacity of human macrophages and T cells for mRNA uptake and translation relative to other cell types tested.

### Comparison of cancer cell clearance by CAR-T cell-mediated cytotoxicity and CAR-macrophage-mediated phagocytosis

The subsequent phase of the study involved the examination of the impact of the CAR mRNA transfection on the interactions of CAR-T cells and CAR-macrophages with CD19^+^ NALM6 CAR target cells as compared to CD19^-^ K562 and THP-1 cells, which were used as negative controls (Fig. 3A). We therefore co-cultured NALM6 leukemia cells with CAR-T cells and performed a cytotoxicity assay for 24 h (Fig. 3B). To this end, NALM6 cells were fluorescently labelled (CellTracker™ Deep Red, CTR, see details in Table 2) to facilitate their identification in flow cytometric analysis (Fig. 3C). We assessed the cytotoxic effects of the CAR-T cells using flow cytometry across a range of effector-to-target (E:T) ratios: 1:20, 1:10, 1:5, 1:1, 5:1, and 10:1 (Fig. 3D). The killing efficiency increased proportionally with the E:T ratio, ranging from 33.47% dead cancer cells at an E:T ratio of 1:20 to 86.33% at a ratio of 10:1 (Fig. 3E). CAR-T cells eliminated CD19^±^ cancer cells significantly more effectively than CD19^-^ cells (Fig. S7A). At an E:T ratio of 1:1, the CAR-T cells eliminated 82.2% of the CD19^+^ NALM6, which was significantly higher than the percentages for the K562 (32.7%) and THP-1 (43.8%) (Fig. S7B).

**Fig. 3:**
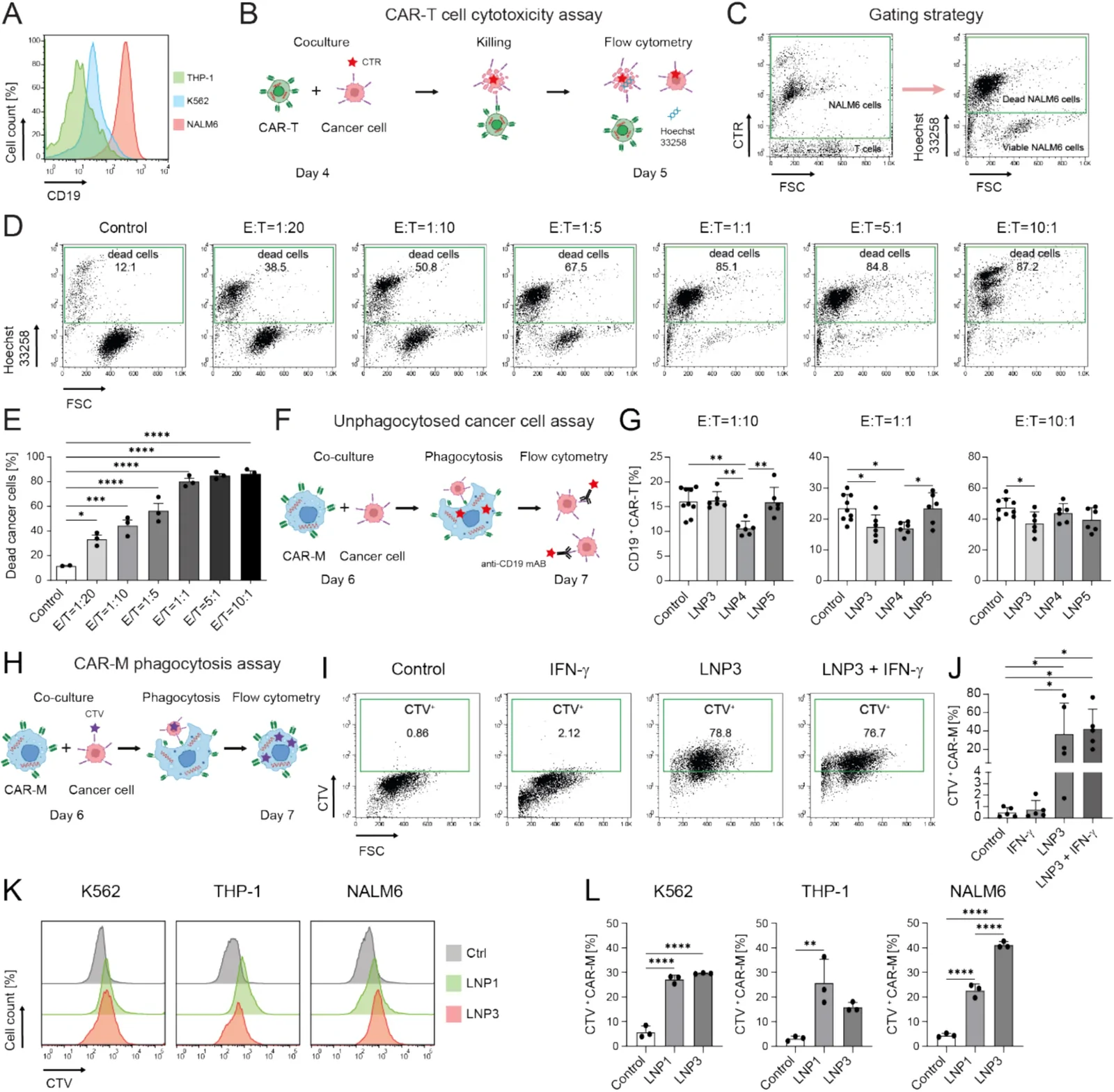
**Comparison of CAR-T cell-mediated killing and CAR-macrophage-mediated phagocytosis of cancer cells.** (A) Comparison of the CD19 signal between the three leukemia cell lines THP-1 (CD19^-^), K562 (CD19^-^), and NALM6 (CD19^+^). (B) Schematic of the CAR-T cell cytotoxicity assay. (C) Gating strategy for the assay shown in B. (D) Representative flow cytometric plots illustrating killing efficiency of CAR-T cells across various E:T ratios, and (E) statistical evaluation of cancer cell viability [one-way ANOVA with Tukey’s post hoc test, n = 3]. (F) Scheme of NALM6 cells co-culture with CAR-M with the readout of unphagocytosed NALM6 cells, and (G) flow cytometric quantification of the percentage of CD19^+^ cancer cells in the supernatant of the co-culture system, using different E:T ratios and two different types of CAR mRNA-LNP (LNP3 and LNP4) or control RNA-LNP (MicroRNA 26b, LNP5, 2 µg/mL) [one-way ANOVA with Tukey’s post hoc test, n = 6–9]. (H) Schematic of the CAR-M phagocytosis assay based on incubating CAR-M with CTV-stained NALM6. (I) Flow cytometric dot plots of one CAR-M donor treated with either IFN-γ (100 ng/mL), LNP3, or LNP3 + IFN-γ (100 ng/mL), prior to co-culture with CTV-stained NALM6 (E:T ratio 10:1), (J) statistical evaluation of the data shown in I [one-way ANOVA with Tukey’s post hoc test, n = 5]. (K) CTV signal of untransfected macrophages, LNP1-treated macrophages, or CAR-M after 24 h of co-culture with various CTV-stained NALM6, K562, or THP-1 cells, and (L) statistical analysis thereof [one-way ANOVA with Tukey’s post hoc test, n = 3]. * *p* ≤ 0.05, ** *p* ≤ 0.01, *** *p* ≤ 0.001, **** *p* ≤ 0.0001.

Subsequently, we sought to ascertain whether the phagocytic engulfment of cancer cells by CAR-macrophages would prove to be as efficacious as the cell death induced by CAR-T cells. Therefore, we determined the reduction of floating NALM6 cancer cells after co-incubation with human CAR-M at selected E:T ratios. Flow cytometry was done to quantify the cancer cells that were not phagocytized and hence remained in the supernatant (Fig. 3F). Across all E:T ratios, LNP3 and LNP4 induced comparable reductions in CD19^+^ cancer cells. At an E:T ratio of 1:10, LNP4 showed the greatest activity, decreasing CD19^+^ cancer cells by 5.42% relative to the control. At 1:1, both formulations mediated similar reductions (6.09% and 6.59% for LNP3 and LNP4, respectively), exceeding the effect of LNP5 containing microRNA-26. At the highest E:T ratio of 10:1, LNP3 sustained a 12.61% reduction in CD19^+^ cancer cells compared with the untreated control (Fig. 3G).

Since we observed a decrease in the percentage of CD19^+^ cells after co-culture with CAR-M, we conducted a follow-up experiment to investigate whether CAR-M affected cancer cell viability − potentially through cytokine secretion − by quantifying the viability of the NALM6 cells that remained in the co-culture supernatant (Fig. S8A). PI staining revealed comparable proportions of dead cancer cells between the untreated control group and the LNP-treated groups (Fig. S8B). Despite the LNP3 group demonstrating the highest number of dead cancer cells, the statistical analysis revealed no significant differences between the three groups (Fig. S8C). This experiment demonstrated that the observed reduction in the percentage of CD19^+^ cells in Fig. 3G was not primarily attributable to direct macrophage-mediated killing, but rather was a result of phagocytosis.

To investigate the reason for the reduction of the cancer cell count in the CAR mRNA-LNP group, we labeled the NALM6 cells with CellTrace™ Violet (CTV, Table 2) before conducting the phagocytosis assay (Fig. 3H). We studied the impact of CTV and other probes on the viability of the cancer cells in monocultures (Table S2). A significantly higher portion of macrophages was CTV^+^ when they were treated with LNP3 and LNP3 combined with interferon-gamma (IFN-γ) (Fig. 3I). Statistical analyses demonstrated that 0.5% of macrophages in the control group and 0.74% in the interferon-gamma (IFN-γ) treatment group were CTV^+^. In contrast, the LNP3 group and the LNP3 + IFN-γ group showed significantly higher percentages of CTV^+^ cells, with 36.71% and 42.16%, respectively, as compared to the control groups (Fig. 3J). We studied five different donors and illustrate their differences in cancer cell phagocytosis apparent in flow cytometric dot plots (Fig. S9).

To study the specificity of phagocytosis of cancer cells by CAR-M and control macrophages transfected with LNP1 further, we incubated them with the CD19^±^ NALM6 cells, or CD19^-^ K562 or THP-1 cells at an E:T ratio of 10:1. Although LNP1 generally increased the uptake of cancer cells by macrophages, CAR-M phagocytosis was most strongly induced upon incubation with NALM6 cells compared to macrophages treated with LNP1 (Fig. 3K). Statistical evaluations demonstrated that only this induction was statistically significant when comparing LNP1 to LNP3 (Fig. 3L).

Importantly, to confirm that the CAR-macrophages phagocytosed viable cancer cells upon CAR transfection, we assessed the viability of CTV-stained cancer cells, and demonstrated that, of all cells scoring CTV^+^ (Fig. S10A), only a few percent were dead (Fig. S10B), with no statistically significant effects on cell viability (Fig. S10C).

These data suggest that CAR-macrophages eliminate cancer cells through phagocytosis, with a significantly lower efficiency than the killing of cancer cells by CAR-T cells.

### Dynamics of CAR-macrophage phagocytosis of cancer cells

To elucidate the longitudinal cellular processes underlying the phagocytosis of cancer cells by CAR-M, we next employed a live-cell imaging approach. NALM6 cells were labelled with CellTracker™ Deep Red (CTR) or with an siRNA bearing a 3′ Alexa Fluor 647 label encapsulated into LNP (SIR) and co-cultured with CAR macrophages (Fig. 4A), followed by analysis of phagocytosis and motility (Fig. 4B). To assess the effects of CTR labelling on cancer cell viability, we conducted flow cytometric analysis (Fig. S11A). Our analyses revealed that CTR staining, across a range of concentrations (250 nM to 2000 nM), did not affect CTR + cancer cell viability (Fig. S11B). We further analyzed the impact of CTR and of all other cell trackers on NALM6 viability using Annexin V/PI staining, revealing that the dyes did not significantly reduce cell viability (Table S2).

**Fig. 4:**
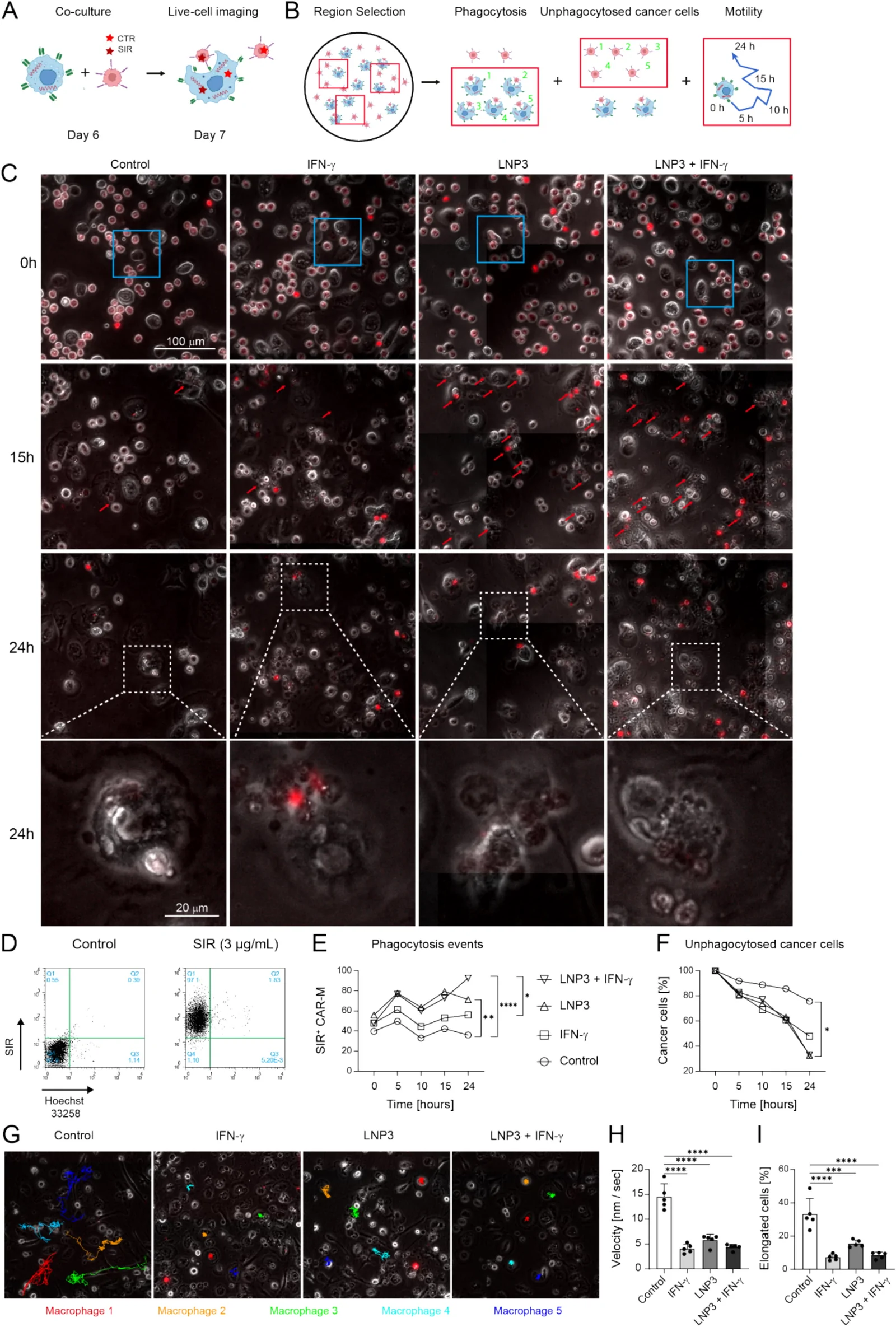
**Macrophage phagocytosis of cancer cells monitored via live-cell imaging.** (A) Schematic representation of live-cell imaging of CAR-M phagocytosis of NALM6 cells labeled with CellTracker™ Deep Red (CTR; see Table 2) or LNP-encapsulated negative control siRNA conjugated with a 3′ Alexa Fluor 647 label (SIR; see Table 2). (B) Selected regions in time-lapse movies were analyzed for phagocytosis and cellular motility by compiling movie stills into sequential frames. (C) Selected stills of live-cell imaging of NALM6 labelled with CTR and co-cultured with macrophages at an E:T ratio of 1:1. Blue rectangles mark selections for movies from four distinct macrophages treatment conditions (compiled in M**ovie S1). Red arrows indicate phagocytosis events observed after 15 h and white dashed rectangles highlight zoom-ins of phagocytosis events. (D) Flow cytometric analysis of NALM6 cells viability: unstained (control) versus stained with SIR and viability staining using Hoechst 33258. (E) Quantification of phagocytosis events in live-cell imaging and (F) unphagocytosed cancer cells in which SIR-labeled NALM6 were co-cultured with macrophages at an E:T ratio of 5:1 and were either untreated (control), treated with 2 µg/mL of LNP3, 100 ng/mL IFN-γ, or LNP3 + 100 ng/mL IFN-γ [two-way ANOVA with Tukey’s post hoc test, n = 3]. (G) Visualization of macrophage motility over a 24-hour period referring from the live-cell imaging experiment shown in Movie S3. (H) Statistical analysis of CAR-M velocity and (I) morphology [one-way ANOVA with Tukey’s post hoc test, n = 5]. * *p* ≤ 0.05, ** *p* ≤ 0.01, *** *p* ≤ 0.001, **** *p* ≤ 0.0001.

Live-cell imaging demonstrated a marked increase in phagocytic activity upon CAR mRNA transfection: in both the LNP3 and LNP3 + IFN-γ groups, macrophages exhibited active engulfment of cancer cells, while macrophages in the control and IFN-γ groups showed no uptake of cancer cells, even when these were in close proximity (Fig. 4C, **Movie S1**). After 15 h, significant phagocytosis events were observed in the LNP3 and LNP3 + IFN-γ groups. By 24 h, macrophages treated under these conditions simultaneously phagocytosed multiple NALM6 cells in some cases (Fig. 4C).

This dynamic visualization confirmed not only the clearance of cancer cells by CAR-M, but also their capability to engulf multiple cells. In subsequent experiments, cancer cells were also labeled with the dye CellTracker™ Deep Red (CTR, Table 2). Quantification of the remaining cancer cells in the supernatant after the phagocytosis assay revealed that the LNP3 and the LNP3 + IFN-γ group exhibited significantly lower proportions of unphagocytosed cancer cells at E:T ratios of 10:1 (Fig. S11C) and 5:1 (Fig. S11D) compared to their respective control groups.

The third strategy to label the cancer cells for detection in flow cytometric assays was to label them with siRNA-LNP (SIR) (Table 2). After 24 h of exposure to 3 µg/mL SIR, only 1.83% of the cancer cells were non-viable (Fig. 4D). Lower concentrations of SIR resulted in an even smaller percentage of dead cells (Fig. S12A). Extended monitoring over 48 h confirmed the minimal impact on cell death (Fig. S12B). Live-cell imaging with SIR at a 5:1 E:T ratio produced results consistent with CTR labeling, with only macrophages having actively phagocytosed cancer cells in the LNP3 and LNP3 + IFN-γ groups as shown in movie stills in Fig. S13, and in Movie S2. After 15 h, phagocytosis events were clearly visible in the LNP3 and LNP3 + IFN-γ groups. Zooming in on the regions at 24 h showed macrophages engulfing NALM6 cells (Fig. S13).

Quantitative analysis of cancer cell counts and phagocytosis frequencies at 0, 5, 10, 15, and 24 h revealed that treatment with LNP3, with or without additional IFN-γ, resulted in an elevated number of SIR^+^ CAR-M (Fig. 4E) and significantly fewer unphagocytosed cancer cells compared to the control group (Fig. 4F). Macrophage motility was quantified using live-cell imaging over 24 h (Fig. 4G**, Movie S3**). Notably, macrophages in the control group exhibited significantly higher motility than those in the IFN-γ, LNP3, or LNP3 + IFN-γ groups (Fig. 4H). Increased mobility upon CAR transfection was associated with fewer cells exhibiting an elongated morphology (Fig. 4I).

These results imply that CAR mRNA and IFN-γ stimulation alter macrophage morphology and movement patterns. These changes in motility and shape may indicate the enhanced phagocytic activity observed in LNP3 and LNP3 + IFN-γ macrophages. Together, these results highlight the complex interplay between cell behavior and cancer cell clearance.

### Effects of CAR mRNA on the polarization of CAR-macrophages

To investigate the impact of the CAR mRNA-LNP on macrophage polarization, we conducted a qPCR analysis on genes associated with M1 or M2 macrophages. Our findings indicate that cells treated with LNP3 or LNP3 + IFN-γ exhibited a substantial upregulation of *Tumor Necrosis Factor* (*TNF*) gene transcription, a pivotal M1 marker. In contrast, IFN-γ alone did not result in elevated *TNF* levels. Although LNP3 led to an increase in *TNF* transcription, another M1 marker, namely *Interleukin-1 beta* (*IL-1B*), remained unaltered. Interestingly, both LNP3 and LNP3 + IFN-γ reduced the transcription of the *C*–*C motif Chemokine Ligand 22* (*CCL22*), an M2 marker, compared to IFN-γ alone. Furthermore, LNP3, IFN-γ, and the combination of both increased *Transforming Growth Factor-beta* (*TGFB*) gene transcription independently (Fig. 5A).

**Fig. 5:**
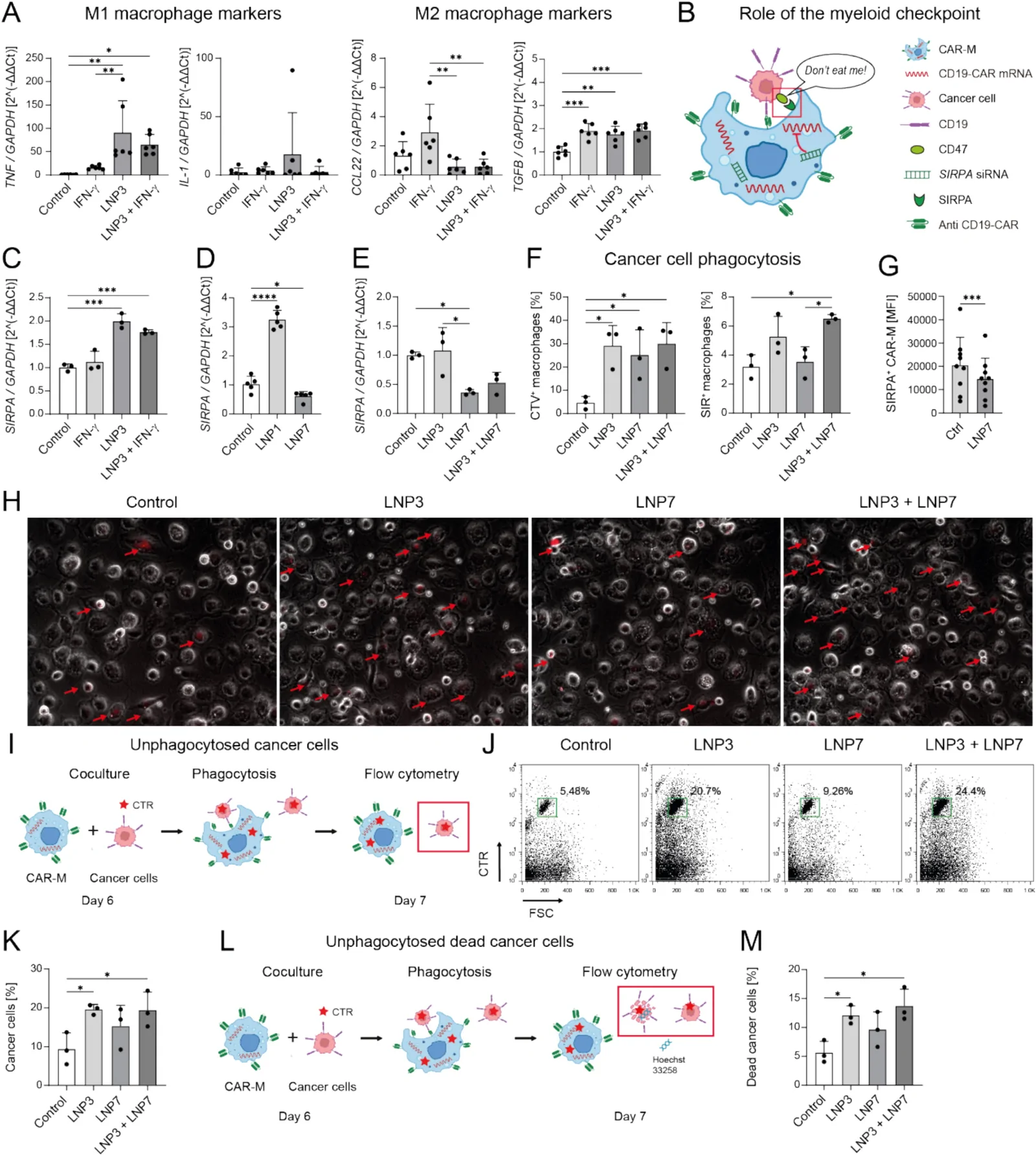
**CAR mRNA-LNP induce a distinct macrophage phenotype and lead to upregulation of *SIRPA* by CAR-M** (A) Gene expression of selected M1 and M2 macrophage markers in macrophages treated as shown in Fig. 4E [one-way ANOVA with Tukey’s post hoc test, n = 6]. (B) Scheme of the myeloid checkpoint illustrating the impact of the combination of CAR mRNA and *SIRPA* siRNA on the CD47-SIRPA axis. (C) *SIRPA* gene expression in macrophages upon treatment with 100 ng/mL IFN-γ, 2 µg/mL of CAR mRNA LNP (LNP3), or both [one-way ANOVA with Tukey’s post hoc test, n = 3]. (D) Impact of 2 µg/mL *Egfp* mRNA-LNP (LNP1) or 1 µg/mL of *SIRPA* siRNA-LNP (LNP7) on *SIRPA* gene expression in macrophages [one-way ANOVA with Tukey’s post hoc test, n = 5]. (E) Effects of 1 µg/mL of *SIRPA* siRNA LNP (LNP7), 2 µg/mL of LNP3, and their combination, on *SIRPA* gene expression in macrophages [one-way ANOVA with Tukey’s post hoc test, n = 3]. (F) Analysis of CAR-M phagocytosis of CTV or SIR-labeled cancer cells [one-way ANOVA with Tukey’s post hoc test, n = 3]. (G) SIRPA surface protein expression after 24 h of treating CAR-M with 1 µg/mL of LNP7 [two-tailed paired Student’s *t*-test, n = 9]. (H) Fluorescence microscopy images showing internalized SIR (red signals) in macrophages following phagocytosis assay. (I) Scheme of the assay to count remaining NALM6 cells after phagocytosis assay (E:T ratio 10:1) and representative flow cytometric plots (J), with corresponding statistical analysis shown in (K) [one-way ANOVA with Tukey’s post-test, n = 3]. Macrophages were treated for 24 h with 2 µg/mL of LNP3, 1 µg/mL of LNP7, or both. (L) Assay scheme for tracking dead NALM6 cells in the supernatant of the co-culture system after phagocytosis assay. (M) Statistical evaluations of the assay scheme presented in (L) [one-way ANOVA with Tukey’s post-test, n = 3]. * *p* ≤ 0.05, ** *p* ≤ 0.01, *** *p* ≤ 0.001.

These findings demonstrate that CAR mRNA-LNP exhibited distinct effects on macrophage polarization and affected both M1 and M2 markers.

### Role of the myeloid checkpoint on cancer cell phagocytosis by CAR-macrophages

In addition to the established role of CAR transduction or transfection in modulating macrophage polarization, we investigated the potential impact of CAR mRNA on the CD47-SIRPA axis (Fig. 5B). Transfection with CAR mRNA-LNP (LNP3) and LNP3 + IFN-γ, but not IFN-γ alone, significantly upregulated *SIRPA* gene expression in macrophages **(**Fig. 5C**)**. To determine whether this induction reflected a broader cellular response to mRNA delivery, we treated macrophages with 2 µg/mL of *Egfp* mRNA-LNP (LNP1), which elicited an even greater, threefold increase in SIRPA expression. To counteract this potentially harmful effect, we further transfected macrophages with *SIRPA* siRNA-LNP (LNP7), which significantly reduced *SIRPA* transcription (Fig. 5D). Remarkably, also the combination of LNP3 with LNP7 effectively counteracted the *SIRPA* upregulation induced by LNP3 (Fig. 5E). The expression analysis of additional genes demonstrated that LNP7, but not LNP3 or LNP3 + LNP7, lead to significantly upregulated *Protein tyrosine phosphatase non-receptor type 11 (PTPN11). Spleen tyrosine kinase (SYK) was significantly upregulated by LNP7 as compared to LNP3.* LNP7 further induced a statistically significant upregulation of *Phosphatidylinositol-4,5-bisphosphate 3-kinase catalytic subunit delta (PIK3CD)* as compared to untreated control cells (Fig. S14).

To investigate the impact of *SIRPA* inhibition on the uptake of cancer cells by CAR-M, we co-cultured CTV-labeled cancer cells with macrophages that had been treated with either LNP3, LNP7, or both. Our findings indicate that each treatment significantly increased phagocytosis compared to the untreated control group. Interestingly, in a subsequent experiment resembling the scheme shown in Fig. 3H, we observed elevated phagocytosis when using SIR-labeled cancer cells with the combination of LNP3 and LNP7 (Fig. 5F). We then investigated the impact of LNP7 on SIRPA surface expression by CAR-M using flow cytometry and detected a significant reduction (Fig. 5G). Fluorescence microscopy revealed that the macrophages treated with LNP3 + LNP7 exhibited the highest SIR-signals following the removal of the cell culture medium, followed by those that received LNP3 (Fig. 5H).

The subsequent objective was to quantify the number of unphagocytosed cancer cells remaining in the co-cultures, through binding to CAR-M (Fig. 5I). Flow cytometric analysis demonstrated that *SIRPA* inhibition using LNP7 slightly enhanced sticking of cancer cells to CAR-M, whereas LNP3 with and without LNP7 led to significantly higher amounts of unphagocytosed cancer cells that stuck to the CAR-M (Fig. 5J), as supported by statistical analysis (Fig. 5K). Next, we determined the number of dead cancer cells binding to CAR-M at an E:T ratio of 10:1 using the viability dye Hoechst 33,258 (Fig. 5L). Both the LNP3 and LNP3 + LNP7 groups exhibited a significantly higher percentage of dead cancer cells compared to the control group (Fig. 5M).

These findings highlight the potential to further enhance CAR mRNA macrophage efficiency by inhibiting SIRPA with siRNA, prompting the question of how cancer cell viability influences phagocytic uptake by CAR‑M.

We then investigated the extent to which apoptosis of cancer cells influences CAR-M−mediated phagocytosis. To explore this, we utilized two methods for inducing apoptosis in the NALM6 cancer cells: 60 min at 45 °C and 60 min at 45 °C with the addition of 3% EtOH. Our findings indicate that incubation at 45 °C for 60 min leads to an average apoptotic rate of 44.97%, with the majority of cells exhibiting early apoptosis (A5^+^PI^-^) characteristics. The addition of 3% EtOH during hyperthermia resulted in the induction of predominantly late-stage A5^+^PI^+^ apoptotic cells (Fig. 6A). However, it is noteworthy that both treatment regimens resulted in a significant number of apoptotic cells (Fig. 6B). The hyperthermia treatment alone resulted in a remaining number of 24.07% apoptotic cells, and the addition of 3% EtOH after an additional 24-hour incubation at 37 °C led to a slightly lower number of apoptotic cells (Fig. S15). We therefore chose to induce apoptosis by hyperthermia induction, with 60 min of incubation at 45 °C for the upcoming experiments.

**Fig. 6:**
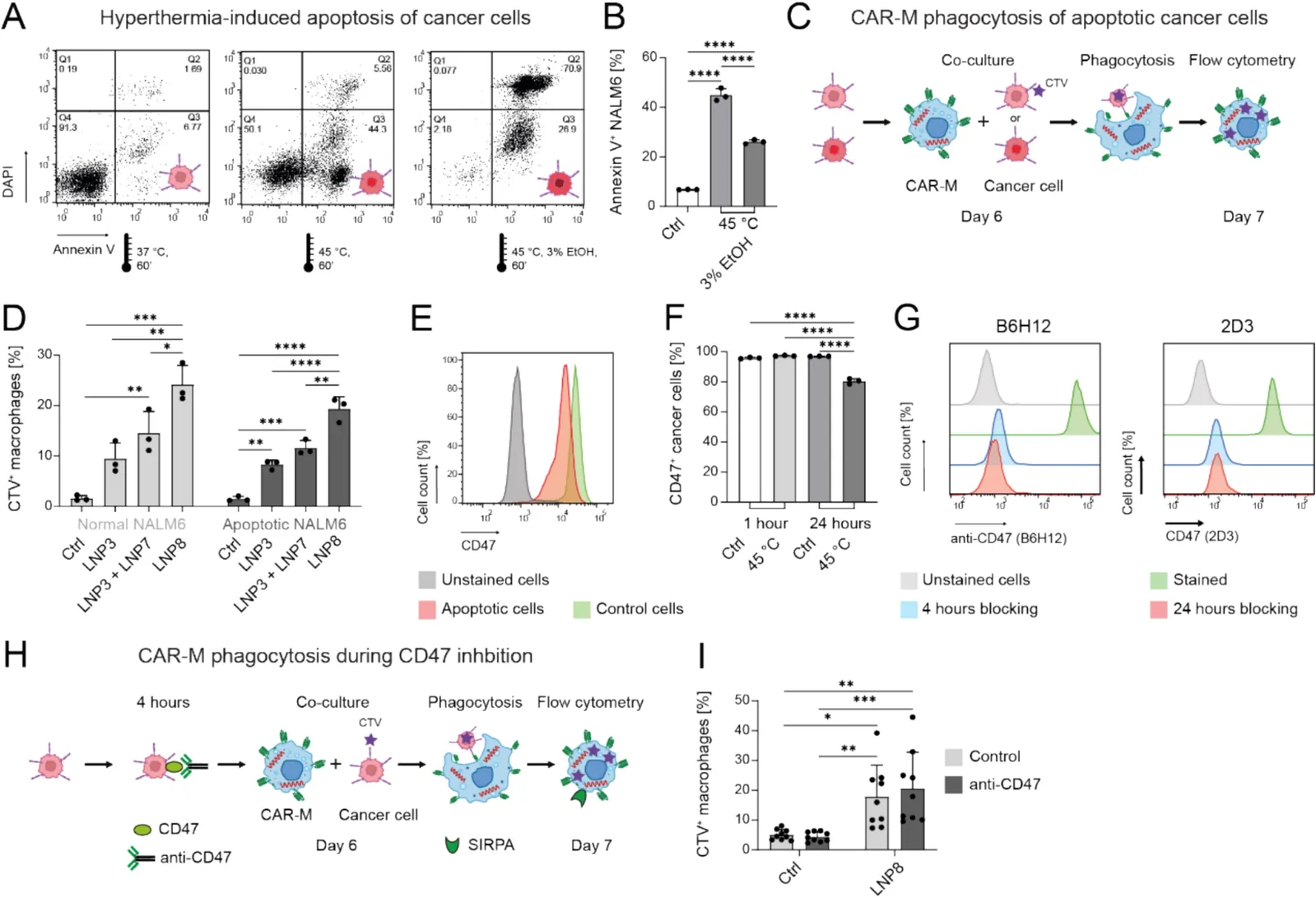
**Impact of cancer cell apoptosis induction on their uptake by CAR-macrophages.** (A) Flow cytometric plots of NALM6 cells exposed to hyperthermia for 60 min at 45 °C with or without 3% ethanol and analyzed by Annexin V/DAPI staining, and (B) statistical analysis of the data [one-way ANOVA with Tukey’s post-test, n = 3]. (C) Scheme showing the uptake of untreated or apoptotic NALM6 cancer cells by macrophages. (D) Statistical data of the assay shown in C, with 3 µg/mL of LNP8 [one-way ANOVA with Tukey’s post-test, n = 3]. (E) CD47 expression of unstained, untreated or apoptotic NALM6 cells, and (F) statistical analysis of the data shown in E [one-way ANOVA with Tukey’s post-test, n = 3]. (G) CD47 surface expression of NALM6 cells either left untreated as controls, or blocked with anti-CD47 antibodies (left panel is clone B6H12 and right panel is clone 2D3) for four or 24 h. (H) Scheme of CAR-M phagocytosis assay with CD47-blocked NALM6 cells, and (I) results of n = 9 human donors derived from flow cytometry [one-way ANOVA with Tukey’s post-test, n = 9]. * *p* ≤ 0.05, ** *p* ≤ 0.01, *** *p* ≤ 0.001.

The next objective was to determine whether induction of apoptosis in NALM6 would affect their phagocytic uptake by CAR-M. To this end, we added apoptotic NALM6 and labeled them with CTV (Fig. 6C). Surprisingly, we found that apoptosis had a low impact on the phagocytic uptake by CAR-M. However, when comparing combined single-agent formulations − CAR LNP alone plus *SIRPA* siRNA LNP alone − using a co-encapsulated formulation (LNP8), we observed significantly enhanced phagocytosis (Fig. 6D). Given the established correlation between apoptosis induction and reduced CD47 expression, we investigated the CD47 expression of apoptotic NALM6 cells. Flow cytometric analysis demonstrated that apoptotic NALM6 cells expressed lower levels of CD47 after 24 h, with an average of 80.4% of the cells showing reduced CD47 expression, which was statistically significant (Fig. 6E,F).

As indicated by previous studies, CD47 blockade has been shown to enhance macrophage-mediated phagocytosis [23]. Therefore, we treated NALM6 cells with two different CD47-blocking antibodies, which reduced CD47 surface expression by approximately 70% after 4 h (Fig. 6G). The subsequent assay therefore sought to determine whether blocking CD47 would impact the uptake of the NALM6 cells by CAR-M (Fig. 6H). Interestingly, we noted a modest increase in cancer cell uptake by CAR-M upon CD47 blocking (Fig. 6I).

Collectively, these findings indicate that modulating *SIRPA* expression enhances the capacity of macrophages to eliminate cancer cells by phagocytosis.

### Analysis of the interactions between CAR-macrophages and CAR-T cells on the elimination of leukemia cells

We then co-cultured CAR-T cells, CAR-M, and NALM6 leukemia cells in a triple cell co-culture using live-cell imaging for up to 24 h (Fig. 7A). In the control group (upper panel), the three cell types remained clearly separated, whereas we noted a higher number of cell–cell contacts upon CAR transfection. Specifically, CAR-M engulfed cancer cells, and T cells interacted more frequently with CAR-M (Fig. 7B). Macrophages in the control group exhibited only negligible phagocytic activity and also the T cells did not interact with cancer cells. In contrast, CAR-M and CAR-T cells retained cancer cells attached to their surface, in some cases, multiple cancer cells bound to a single CAR-M (**Movie S4**).

**Fig. 7:**
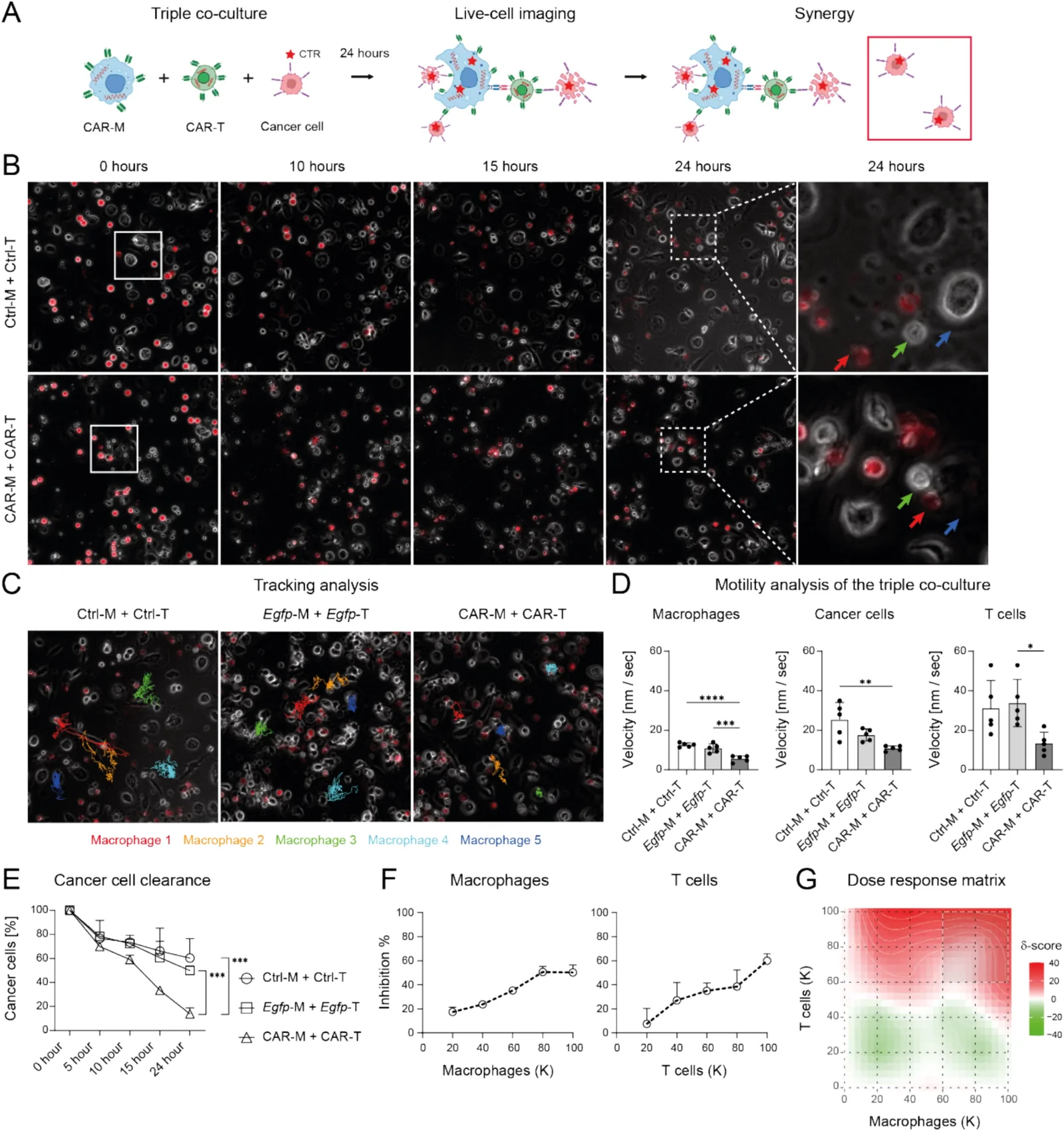
**Motility and synergistic inhibitory effects of CAR-macrophages and CAR-T cells.** (A) Scheme of live-cell imaging of a triple co-culture system comprising macrophages, T cells, and NALM6 cancer cells. CAR-M and CAR-T cells were generated in parallel using LNP3. CAR-T cells and CTR-labelled NALM6 cells were added to CAR-M and live-cell imaging was done. (B) Live-cell imaging-derived stills of the triple co-culture system with untransfected cells (Ctrl-M + Ctrl-T), and CAR effector cells (CAR-M + CAR-T). White rectangles correspond to regions shown in Movie S4. Dashed white rectangles highlight zoomed-in regions with red arrows denoting cancer cells, green arrows T cells, and blue arrows macrophages. (C) Stills from Movie S5 showing macrophage tracking analysis over 24 h with different colors distinguishing individual macrophages. LNP1 were used to create an additional control group (*Egfp*-M; *Egfp*-T). (D) Statistical analysis of the velocity data shown in item C [one-way ANOVA with Tukey’s post-test, n = 5]. (E) Quantification of cancer cell elimination in the control cells, LNP1, or CAR effector cells in the triple co-culture system [two-way ANOVA with Tukey’s post hoc test, n = 3]. (F) Inhibition rate of single-agent CAR-T cells or CAR-M alone at different E:T ratios. (G) Synergy analysis using the Loewe synergy model, with grey areas indicating the most synergistic regions. * *p* ≤ 0.05, ** *p* ≤ 0.01, **** *p* ≤ 0.0001.

To unravel if the effects of the CAR transfection are owed to the mRNA transfection *per se* or if it leads to specific cellular changes, and to further study the impact on cell motility, we further added a control group in which T cells and macrophages were treated with LNP1. The motility of the three cell types was quantified in a live-cell imaging experiment (Fig. 7C**, Movie S5**). Statistical quantifications of motility demonstrated that the velocity of macrophages over a 24-hour period was 12.55 nm/sec in the control group, decreased modestly to 10.87 nm/sec in the LNP1 group, and was further reduced to 5.58 nm/sec in the LNP3 group. The mean velocity of cancer cells declined from 25.40 nm/sec in the control group to 17.60 nm/sec following LNP1 treatment, and it further decreased to 10.80 nm/sec upon LNP3 transfection. T cell motility followed the same trend, with velocities of 31.20 nm/sec in the control group, 33.80 nm/sec in the LNP1 group, and a marked reduction to 13.40 nm/sec in the LNP3 group. Statistical analysis confirmed that transfection with CAR mRNA significantly reduced the motility of macrophages, cancer cells, and T cells compared with both untreated and *Egfp* mRNA controls (Fig. 7D). Furthermore, the percentage of residual cancer cells in the co-culture system was quantitatively assessed over time. Our analyses revealed that, when both macrophages and T cells were transfected with CAR mRNA, a mean of 13.87% of cancer cells remained after 24 h, which was significantly lower compared to the control group, where a mean of 60.26% of cancer cells were left (Fig. 7E).

To determine whether the combined activity of CAR-T cells and CAR-M represented valid synergy rather than a simple additive effect, we performed synergy assays. As shown in Fig. 7F, each effector exhibited an inhibitory effect when applied individually. We used a range of 36 different combinations of CAR-T cell and CAR-M cell numbers from three different donors (Table S4). However, when both were used in combination, data analysis using the Loewe additivity model revealed an average synergy score of 10.013 ± 2.97, exceeding the critical threshold of 10, and indicating synergistic interaction between CAR-T cells and CAR-macrophages. Notably, the highest synergy score of the dose matrix was 21.05 (Fig. 7G).

These data demonstrate that CAR-T and CAR-M actually exhibit synergistic, but not additive effects.

## Discussion

Our findings demonstrate that LNP-based transfection is better tolerated by T cells compared to EP, consistent with observations reported by other groups [24], [25], [26]. Notably, our study provides new insights into the role of the LDLR, which serves as the entry point for LNP uptake in various cell types, especially hepatocytes [27]. The induction of LDLR expression in T cells upon their activation, as observed in this study, may share mechanistic parallels with previously described cholesterol-induced upregulation of LDLR gene expression in T cells. This suggests a conserved regulatory pathway influencing LNP internalization [28].

Previous studies investigating the cytotoxicity of CAR-T cells have generally reported that E:T ratios of 1:1 or higher are required to achieve significant elimination of cancer cells [29], [30], [31]. In contrast, our experiments demonstrated, notable effects on cancer cell viability even at a much lower E:T ratio of 1:20. This discrepancy is likely attributable to differences in experimental design and the methodologies employed for assessing target cell viability. Specifically, our study utilized propidium iodide (PI) and Annexin V staining, which are sensitive in detecting early apoptotic and necrotic cell death, whereas other studies employed lactate dehydrogenase (LDH) release assays [28], nucleic imaging combined with CellTiter-Glo® [29], or the ^51^Cr release assay [30], each with distinct sensitivity and detection windows.

In our study, CAR-macrophages demonstrated potent phagocytic activity across various E:T ratios, with peak activity observed at a 10:1 ratio. This observation aligns with the findings reported by Lei *et al.* [32]. The observed differences in cancer cell elimination between mRNA-based CAR T-cells and CAR-M may be attributed to the intrinsically lower migratory capacity of macrophages compared with T cells [33], [34]. Additionally, variability in CAR protein expression levels resulting from the use of different gene delivery vectors across studies could contribute to the observed discrepancies in cancer cell elimination frequencies [29], [35].

Notably, the substantial decrease in CAR-M motility upon CAR mRNA transfection could have significant implications for CAR-M therapy. It is an established fact that CAR-M exhibit a significantly higher capability than CAR-T cells to infiltrate tumors and remodel the tumor environment [13], and mobility is an important prerequisite for the remodeling. To counter the reduced motility of CAR-M observed after transfection, synthetic RNA molecules that restore migration could provide an effective strategy. This could be implemented by using microfluidic co-encapsulation of selected motility-promoting RNAs and CAR mRNA into LNP [16].

The diminished cellular stretching observed in live-cell imaging suggest increased activation of CAR-M, consistent with previous reports [24], [35], [36]. While others, who also employed human primary immune cells, demonstrated that unmodified CAR mRNA − as employed in our system − induces macrophage activation [24], our gene expression analyses revealed features characteristic of both pro-inflammatory and alternative or anti-inflammatory macrophages. This is reflected by CAR mRNA-induced upregulation of inflammatory *TNF* on the one hand, and anti-inflammatory mediators such as *TGFB* expressed by alternatively activated macrophages on the other hand. Interestingly, Kahwaji and colleagues also showed that, upon replacing uridine with nucleoside analogs such as 5-methoxy-uridine (5moU) or N1-methyl-pseudouridine (m1Ψ), CAR-M activation was significantly reduced [24]. Notably, these uridine derivatives did not affect CAR-T cell activation [24], which is very likely owed to the fact that T cells exhibit significantly lower levels of Toll-like receptors (TLR) which recognize nucleic acids, specifically TLR7 and TLR8 [37].

The activation of CAR-M and the related induction of SIRPA may be a general feature of unmodified mRNA [24], because all types of CAR mRNA and also *Egfp* mRNA and induced its upregulation, while the siRNA-LNP and IFN-γ did not. The co-transfection of *SIRPA* siRNA and CAR mRNA-LNP effectively counteracted *SIRPA* upregulation by CAR and *Egfp* mRNA, while co-encapsulation of CAR mRNA and *SIRPA* siRNA even further potentiated the CAR-M phagocytosis. Morrissey *et al.* demonstrated that *SIRPA* suppresses integrin activation at phagocytic synapses, thereby restricting macrophage spreading across the surface of the engulfment target [15]. Moreover, Zhang and colleagues silenced SIRPA via short hairpin RNA to disrupt CD47-SIRPA signaling [38]. The findings of these studies, in conjunction with our data, suggest that further investigation into the therapeutic blocking of *SIRPA* is warranted.

To distinguish the cancer cells from CAR effector cells, we employed CTV, CTR, and LNP-encapsulated SIR. CTV proved ideal for flow cytometry due to its sharp emission spectrum; however, we avoided it for live-cell imaging because the 405-nm violet excitation light induces strong phototoxicity, which can lead to cellular damage [39]. Instead, we used CTR and SIR in live-cell imaging, as CTR provided prolonged fluorescence stability and brightness, with only moderate effects on cancer cell viability.

Our triple co-culture analyses indicate that human primary CAR-T cells and CAR-M exert synergistic cytotoxicity. The observed synergy likely stems from cytokine-induced reciprocal activation, with CAR mRNA-LNP polarizing macrophages to enhance T-cell cytotoxicity, and *vice versa*. Activated T cells, including CAR-T cells, secrete e.g. IFN-γ which leads to M1 polarization [40], whereas activated CAR-M release mediators such as TNF [13], which drives tumor cell apoptosis and bolsters T cell function. Cell-cell contacts observed in live-cell imaging may accelerate cytokine exchange, without trogocytosis or antigen presentation.

The clearest evidence of synergy between CAR-M and CAR-T cells has been demonstrated in preclinical *in vivo* models of checkpoint blockade [13]. Recently, clinical trials of CT-0508 have demonstrated accumulation and remodelling of CAR-M within the tumor microenvironment, accompanied by an expansion of CD8^±^ T cells [41].

Nevertheless, as with most *in vitro* studies, our work has limitations. Co-culture systems lack the heterogeneous immune cell subsets and tumor microenvironment components encountered *in vivo*. For example, it lacks additional myeloid and lymphoid populations that influence effector function. Furthermore, *in vitro* systems do not replicate systemic circulation, soluble mediators (e.g., serum proteins and complement), or physiological clearance mechanisms that affect the persistence and efficacy of CAR-T cells and CAR-M.

## Conclusion

Our findings highlight the ability of CAR mRNA to modulate immune cell behavior, particularly mediated by reducing macrophage motility and induction of phagocytosis. Our data further suggest that CAR mRNA-LNP treatment not only reduces the motility of macrophages, T cells, and cancer cells, but that it also fosters stronger intercellular interactions through cell-to-cell contacts.

A key insight of our research is the synergistically enhanced cancer cell clearance upon co-culturing CAR-T cells with CAR-M, as compared to monocultures. Additionally, our study underscores the added value of targeting “don’t eat me” signals, such as *SIRPA* downregulation by siRNA, to further optimize macrophage-mediated engulfment of cancer cells.

These findings indicate that dual-cell therapy approaches harnessing the complementary strengths of CAR-T cells and CAR-macrophages merit further exploration to enhance treatment outcomes. Future *in vivo* studies should examine varying cell numbers and ratios, as well as staggered administration of CAR effector cells. These investigations will determine whether the observed synergy enables reduced cell dosing in CAR-T/CAR-macrophage combinations relative to established CAR-T or CAR-macrophage monotherapies, thereby broadening the therapeutic window and minimizing CAR-T cell toxicities. This could pave the way for improved interventions in patients with hematological malignancies and potentially other cancer types.

## Compliance with Ethics Requirements

All procedures followed were in accordance with the ethical standards of the responsible committee on human experimentation (institutional and national) and with the Helsinki Declaration of 1975, as revised in 2008 (5). Informed consent was obtained from all patients for being included in the study.

## Declaration of competing interest

The authors declare that they have no known competing financial interests or personal relationships that could have appeared to influence the work reported in this paper.
